# Elucidating epigenetic mechanisms governing odontogenic differentiation in dental pulp stem cells: an in-depth exploration

**DOI:** 10.3389/fcell.2024.1394582

**Published:** 2024-05-28

**Authors:** Lei Huang, Xuan Chen, Xiaoxia Yang, Yinchun Zhang, Yiyun Liang, Xiaoling Qiu

**Affiliations:** Department of Endodontics, Stomatological Hospital, School of Stomatology, Southern Medical University, Guangzhou, Guangdong, China

**Keywords:** epigenetic regulation, odontogenic differentiation, pulp, DPSC, LncRNA, long noncoding RNA

## Abstract

Epigenetics refers to the mechanisms such as DNA methylation and histone modification that influence gene expression without altering the DNA sequence. These epigenetic modifications can regulate gene transcription, splicing, and stability, thereby impacting cell differentiation, development, and disease occurrence. The formation of dentin is intrinsically linked to the odontogenic differentiation of dental pulp stem cells (DPSCs), which are recognized as the optimal cell source for dentin-pulp regeneration due to their varied odontogenic potential, strong proliferative and angiogenic characteristics, and ready accessibility Numerous studies have demonstrated the critical role of epigenetic regulation in DPSCs differentiation into specific cell types. This review thus provides a comprehensive review of the mechanisms by which epigenetic regulation controls the odontogenesis fate of DPSCs.

## 1 Introduction

Pulpitis refers to the inflammation of the dental pulp, typically caused by bacterial infection, resulting in severe pain (Kakehashi et al., 1965; Brännström and Nyborg, 1973) Over time, the pulp tissue may degenerate, leading to apical periodontitis, osteomyelitis, and eventually necessitating tooth extraction (Shi et al., 2005). However, effective management of pulp infections can stimulate the formation of reparative dentin and protect the pulp from further damage (Tronstad and Mjör, 1972). Among the treatment options, vital pulp therapy (VPT) presents a less invasive and more conservative approach compared to root canal therapy (RCT) (Ferracane et al., 2010), although the inability to accurately assess the severity of pulpitis clinically limits its potential for restorative success (Barthel et al., 2000; Ricucci et al., 2014). As regenerative medicine advances, modulating the expression of specific molecules can effectively suppress inflammation, promote the growth of reparative dentin, and enhance the overall regenerative capacity of the pulp (Gangolli et al., 2019).

Human dental pulp-derived stem cells (hDPSCs) are a type of mesenchymal stem cell developed from neural crest cells, which originate from ectodermal tissue (Nuti et al., 2016). In 2000, Gronthos et al. (2000) were the first to successfully culture colony-like cells from human third molars in 2000. These cells were found capable of differentiating into odontoblast-like cells and forming dentin-like structures, indicating the presence of stem cells in pulp tissue and leading to the initial concept of DPSCs. Subsequently, Miura et al. (2003a) isolated stem cells from the pulp of human exfoliated deciduous teeth (SHEDs), which are similar to mesenchymal stem cells (MSCs) in their undifferentiated or hypodifferentiated state. DPSCs are highly valued for pulp regeneration due to their unique differentiation advantages. Under specific induction conditions, DPSCs can differentiate into various functional cells such as osteoblasts, adipocytes, chondrocytes, odontoblast-like cells, and neuronal cells (Anitua et al., 2018). The odontogenic differentiation of dental pulp stem cells is presently a field that lacks comprehensive study, and the active investigation of the molecular pathways of dentin differentiation, along with the creation of reliable biomarkers, is imperative for examining the formation of the pulp-dentin complex. It has been discovered that chromatin state, transcriptional regulation, and epigenetic modifications collectively perform a part during the self-renewal and differentiation process of DPSCs (Bayarsaihan, 2016). Epigenetics has emerged as a prominent research focus due to its ability to modulate functional genes without altering DNA sequences (Wu and Morris, 2001). Thus, this paper offers both theoretical and empirical support for clinical endodontic practices, especially VPT, by exploring the immunophenotypes and related epigenetic processes that guide DPSCs potential for odontogenic differentiation.

## 2 Epigenetic

**FIGURE 1:**
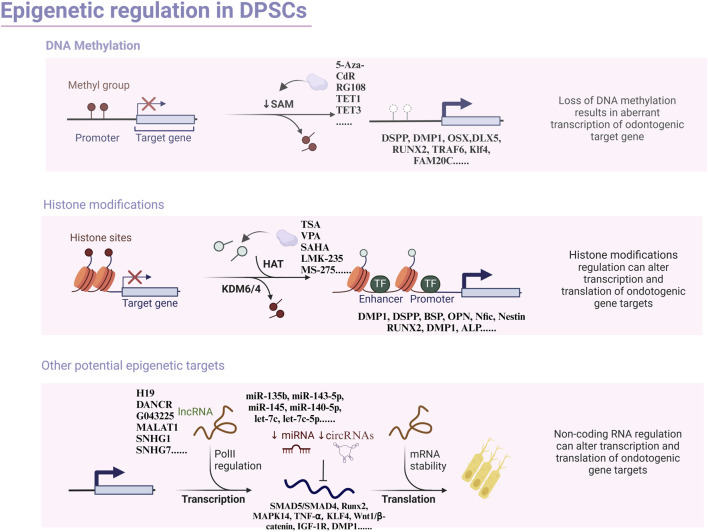
Overview of the principal types of epigenetic modification with focus on odontogenic differentiation in dental pulp stem cells.

Epigenetics is a branch of genetics that gradually took shape in the 1980s to investigate how changes in non-genetic sequences impact gene expression levels (Goldberg et al., 2007). The term was initially introduced by Conrad Waddington in 1942 to illustrate the intricate developmental journey from genotype to phenotype (Waddington, 2012). Research has demonstrated that epigenetic modifications play a critical role in the regulation of embryonic stem cells (ESCs) and DPSCs, particularly in governing their self-renewal and differentiation capabilities (Anitua et al., 2018). The most extensively studied epigenetic mechanisms encompass DNA methylation, histone modifications, and non-coding RNA-mediated regulation of gene transcription (Figure 1). Given the breadth of research on classical modifications in contemporary stem cell studies, we aim to revisit and update the existing research on the epigenetic mechanisms of odontogenic differentiation.

### 2.1 DNA methylation

DNA methylation is a ubiquitous process in eukaryotic cells that serves as an established and inheritable epigenetic marker, influencing chromatin organization and gene transcription. This process entails attaching a methyl group to the 5′carbon atom of cytosine (C) within the cytosine-phosphothio-nyl-guanine (CpG) dinucleotide, creating 5-methylcytosine (5 mC). The methyl donor, S-adenosylmethionine (SAM), provides the necessary methyl group, and DNA methyltransferase (DNMT) enzymes catalyze the reaction. (Radhakrishnan et al., 2011). The primary target of DNA methylation is the CpG island, a genomic region rich in cytosine and guanine (CpG) sequences, primarily located near gene promoters and exon 1. CpG islands are typically unmethylated, but high levels of methylation result in stable gene silencing (Bird, 2002). The regulation of CpG methylation levels is dynamic and controlled by DNA methyltransferase (DNMT) and DNA demethylase (TET) enzymes (Li et al., 2015a; Zhang et al., 2018). The DNMT family comprises DNMT1, DNMT3A, DNMT3B, and DNMT3L, with only DNMTs (−1, -3A, and -3B) involved in DNA methylation, while DNMT-3L lacks enzyme activity (Kareta et al., 2006). The TET family, including TET1, TET2, and TET3, and activation-induced deaminases (AID) are required to demethylate DNA by lowering methylation marks in the course of DNA replication (Bochtler et al., 2017). The expression of CpG genes is regulated through two main mechanisms. First, methyl CpG-binding proteins recognize and bind specifically to methylated CpG regions, attracting transcriptional repression cofactors that suppress DNA transcription (Gao et al., 2014), Second, methylation at CpG sites can physically block the binding of DNA-binding proteins, such as transcription factors, to their target sites, thereby inhibiting transcription (Hlady et al., 2014).

### 2.2 Histone modifications

Histones, which encompass H1, H2A, H2B, H3 as well as H4, are incorporated with DNA to constitute nucleosomes, the fundamental units of chromatin. It has been demonstrated that histones play a vital role in promoting transcription in promoter regions (Lee et al., 2004). Moreover, histones undergo various post-translational protein modifications (PTPM), such as methylation, acetylation, ubiquitination, phosphorylation, succination, and ADP-ribosylation (Jenuwein and Allis, 2001), that change their interactions with DNA and with each other (Venkatesh and Workman, 2015). These modifications are crucial for sustaining chromatin structure, nucleosome stability, and gene expression (Zheng et al., 2014; Lawrence et al., 2016; Bascom and Schlick, 2018; Yi and Kim, 2018). Among all the histone post-translational modifications (HPTMs), methylation and acetylation are the most extensively investigated.

#### 2.2.1 Histone methylation

Histone methylation, a covalent modification, occurs on arginine and lysine residues. This process involves transferring a methyl group from SAM to a lysine or arginine site in the presence of histone methyltransferase (Strahl and Allis, 2000). Typically, histone methylation occurs at the N-terminal end of histone H3 or H4. Arginine can undergo mono- or dimethylation, while lysine can be mono-, di-, or trimethylated (Pedersen and Helin, 2010). Stem cell differentiation processes produce enzymes that modify methylation levels at specific genes, influencing gene expression. Depending on the gene site, methylation can either repress or promote transcription.

#### 2.2.2 Histone acetylation

Histone acetylation is predominantly facilitated by histone acetyltransferases (HAT) and histone deacetylases (HDAC) and typically occurs on lysine residues. This is a reversible chemical reaction where HAT catalyzes the acetylation of histone N-terminal lysine residues, neutralizing the positive charge on histones. This, in turn, reduces the affinity of histones for negatively charged DNA, loosening the chromatin structure and ultimately promoting the binding of transcription factors to DNA for the activation of specific genes (Galvani and Thiriet, 2015). Conversely, HDAC-mediated histone deacetylation can lead to gene silencing, as DNA neutralization by histone binding, condensing the chromatin structure, and inhibiting transcription factor binding (Meier and Brehm, 2014). Our comprehension of the mechanisms underlying alternative forms of histone modifications and the implicated signaling pathways remains constrained, warranting further investigations.

### 2.3 ncRNAs

Non-coding RNAs are synthesized from the reverse transcription of protein-encoding genes and lack an open reading frame. This group includes ribosomal RNA (rRNA), transfer RNA (tRNA), small nuclear RNA (snRNA), small nucleolar RNA (snoRNA), and microRNA (miRNA) (Kaikkonen et al., 2011). Non-coding RNA transcripts outnumber mRNA transcripts, yet they do not translate into proteins; instead, they play a role in epigenetically regulating gene expression (Palazzo and Lee, 2015). Among these, miRNA, a highly conserved non-coding single-stranded RNA consisting of 20–23 nucleotides, is found in eukaryotes and has been a central focus of epigenetics research. MiRNA is transcribed from intergenic DNA sequences as an initial transcript known as pri-mRNA, which is then processed into pre-mRNA by the nuclease Drosha and further refined into mature miRNA by Dicer. (Lee et al., 2003; Liu et al., 2004; Cipriani et al., 2022). The mature miRNA is subsequently incorporated into the AGO family of proteins to form the RNA-induced silencing complex (RISC). miRNAs can bind to the 3′-untranslated region (UTR) of mRNAs, resulting in the degradation or post-transcriptional repression of target mRNAs, thereby modulating gene expression (Ha and Kim, 2014; Seong et al., 2014). Additionally, competing endogenous RNAs (ceRNAs) indirectly regulate downstream targets by competing with miRNAs for binding. Among ceRNAs, circular RNA (circRNA), a family of ncRNAs with a ring-like covalent structure, resists RNA enzyme digestion, making it more stable and less susceptible to degradation (Qu et al., 2015; Guo et al., 2020; Zhang et al., 2020). Furthermore, circRNA is widely distributed in living organisms and exhibits expression specificity and high evolutionary conservation (Jeck et al., 2013). In some instances, the richness of circRNA is more than 10-fold that of the relevant linear mRNA, making it an ideal disease marker and treatment target with important clinical and research implications (Li et al., 2015b; Wang et al., 2016). Long non-coding RNAs (LncRNAs) are a new heterogeneous linear ncRNA with more than 200 nucleotides, and their functions are mainly divided into three aspects: modifying chromosomes for apparent regulation, participation in transcriptional regulation through interaction with transcription factors, and post-transcriptional or translation regulation by affecting mRNA processing. LncRNAs may serve as a biomarker in multiple biological processes (Carpenter et al., 2013; Wang et al., 2013; Legnini et al., 2014; Zhu et al., 2014), and increasing evidence suggests that lncRNAs have a major contribution to the regulation of odontogenic differentiation in hDPSCs.

## 3 Dental pulp stem cells

### 3.1 Discovery and characterization of DPSCs

Stem cells possess the remarkable ability to replicate, renew, and give rise to specific cell types, significantly influencing tissue development, repair, and homeostasis (McNeely et al., 2020) Dental pulp stem cells, categorized as DPSC and SHED depending on their origin, were successfully isolated and identified in 2000 and 2003 (Gronthos et al., 2000; Miura et al., 2003b). Both types exhibit the typical characteristics of MSC. DPSCs were the first MSCs of oral and maxillofacial origin to be isolated, paving the way for subsequent discoveries of similar cells, such as SHED, periodontal stem cells, and apical papilla stem cells (Esposito et al., 2003; Innes, 2010; Guven et al., 2011; Jeong et al., 2014). These stem cells express MSC markers but lack haematopoietic markers (Liu et al., 2015a) However, DPSCs are heterogeneous, with varying marker expressions. Specific cell surface markers enable the isolation of DPSC subpopulations for targeted differentiation in clinical settings. Researchers suggest that CD271+ DPSCs possess greater potential for odontogenic differentiation (Alvarez et al., 2015), while the expression level of CD146 correlates with the functionality of dental pulp stem cells, marking it as a key functional indicator of their efficacy (Ma et al., 2021).

### 3.2 Differentiation and application of DPSCs

**FIGURE 2:**
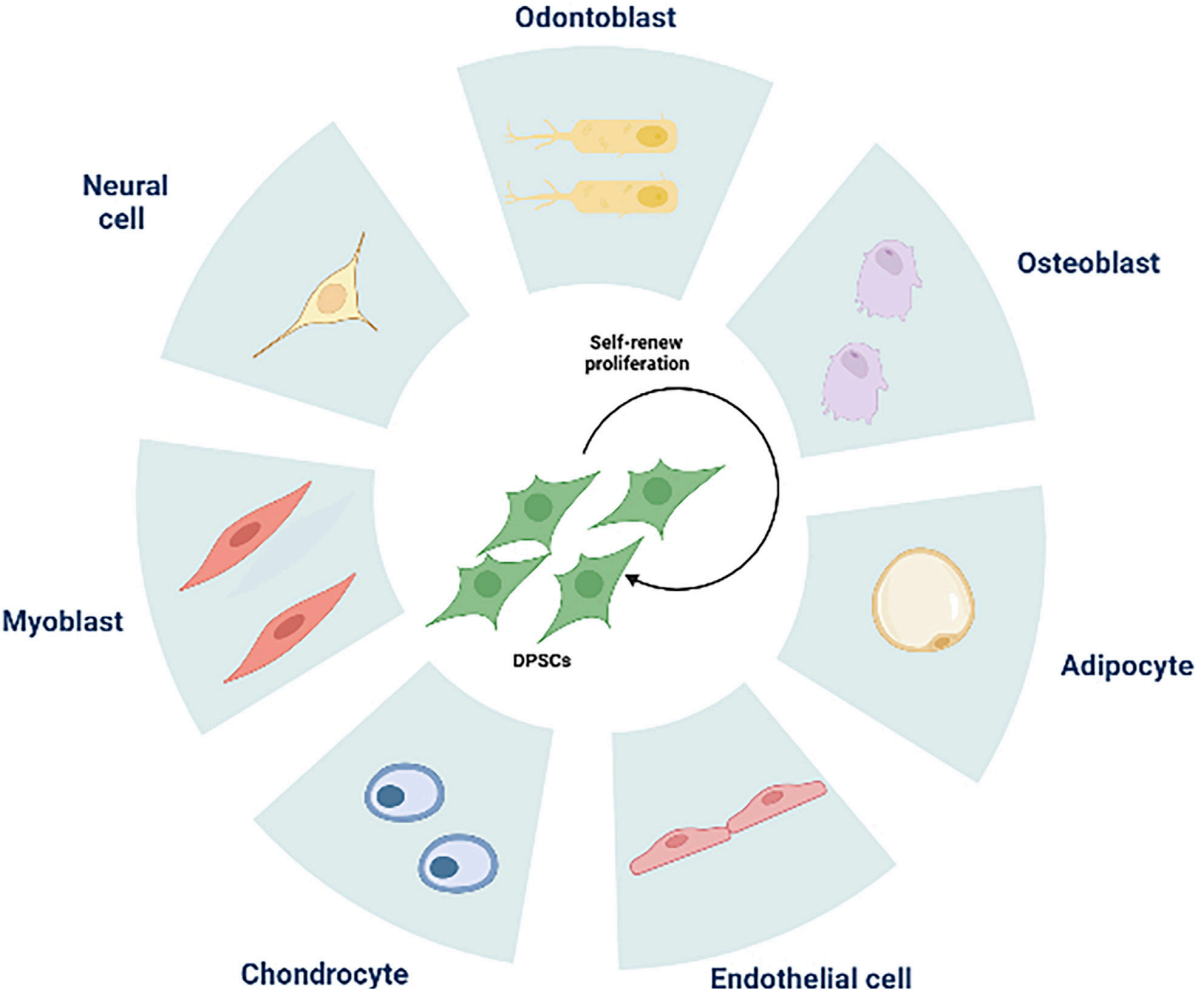
The multilineage differentiation potential of DPSCs. DPSCs can differentiate into odontoblasts, osteoblasts, adipocytes, endothelial cells, chondrocytes, myoblasts, neural cells.

DPSCs share similarities with bone marrow MSCs and express markers related to mineralization and osteogenesis (Chen et al., 2005; Ching et al., 2017). With proper induction, dental pulp stem cells can differentiate into osteoblasts, chondrocytes, adipocytes, and neural-like cells (Zhu et al., 2018) (Figure 2). In a study conducted by Alge et al. (2010), DPSCs demonstrated a higher proliferation rate and greater clonal potential compared to bone marrow MSCs. Lineage tracing in 2014 revealed that DPSCs originate from peripheral nerve-associated glia (Kaukua et al., 2014), which can differentiate into pulp cells in adult mice incisors. Furthermore, the perivascular niche, a distinct environment for various MSCs (Crisan et al., 2008), houses pulp stem cells within the perivascular region of pulp tissue (Shi and Gronthos, 2003). *In vivo* experiments have shown the involvement of dental pulp stem cells in angiogenesis (Iohara et al., 2009; Iohara et al., 2013), demonstrating their strong capacity for promoting pulp regeneration (Yang et al., 2015). In a groundbreaking study conducted in 2000 (Gronthos et al., 2000), Gronthos et al. transplanted DPSC-complexed hydroxyapatite/triple calcium phosphate scaffolds into the dermis of immunodeficient mice, resulting in the formation of pulpal and dentin-like tissues with organized collagen matrices, albeit without haematopoietic or adipocyte formation. This indicates that odontogenic differentiation is a major default phenotype of DPSCs. Furthermore, DPSCs derived from human mammary dental stem cells were integrated with recombinant collagen and injected into the pulp cavity. This integration enabled them to maintain their viability, rebuild vascularized pulp tissues, and differentiate into dentin cells expressing dentin sialophosphoproteins and dentin matrix proteins (Itoh et al., 2018). The diverse and complementary unity of cell populations found in pulp stem cells plays a vital role in repairing and maintaining the local homeostasis of pulp tissues. Achieving functional pulp regeneration through pulp stem cells may rely on the dynamic structure and unity of such diverse cell populations.

## 4 Epigenetic mechanisms

### 4.1 DNA methylation associated with odontogenic differentiation in DPSCs

DNA demethylation activates gene transcription, while DNA methylation typically leads to gene repression. DNA methylation patterns vary among different cell types and contribute to the diverse differentiation potential of stem cells. Through the reprogramming of stem cells, pluripotent stem cells (iPSCs) can be generated for clinical therapy, with DNA methylation playing a pivotal role in this process (Godini et al., 2018) (Table 1). Genome-wide DNA methylation analysis has revealed that the methylation profile of DPSCs closely resembles that of embryonic stem (ES) and induced pluripotent stem (iPS) cells. Specifically, the downregulation of HERV-FRD and upregulation of PAX9 increase the efficacy of DPSCs in differentiating into iPS cells (Dunaway et al., 2017). DNA methylation patterns influence the expression of DPSC genes, with DNA demethylases and DNA methyltransferase (DNMT) activity affecting the differentiation potential of DPSCs. Treatment with the DNA methyltransferase inhibitor 5-aza-2′-deoxycytidine (5-Aza-CdR) reduced the proliferative capacity of DPSCs but upregulated odontogenic markers (DMP1 and DSPP) and transcription factors (OSX, RUNX2, and DLX5), increasing ALP activity and accelerating the formation of calcified nodules, thereby enhancing odontogenic differentiation capacity (Zhang et al., 2015). The novel DNA methyltransferase inhibitor RG108 also regulates DSPP and DMP1, promoting odontogenic differentiation of DPSCs (Sun et al., 2019). Evidence suggests that DNA methylation affects the transactivation of transcription factors (TFs). Kruppel-like factor (KLF4), a critical mediator of cell differentiation and proliferation, undergoes promoter region demethylation during odontoblast differentiation, enabling efficient SP1 binding and transcriptional regulation that upregulates Klf4 expression (Sun et al., 2019). This process promotes odontoblast differentiation and inhibits the growth of primary DPSCs (Lin et al., 2011).

**TABLE 1:** DNA methylation-mediated regulation of odontogenic differentiation in DPSCs.

Epigenetic modifier	Epigenetic mark	Regulatory factors	Odontogenic genes	Result
5-Aza-CdR	DNMT inhibitor	TRAF6	DSPP, DMP1, OSX, DLX5, RUNX2	Odontogenic (+) (Zhang et al., 2015)
RG108	DNMT inhibitor	SP1	Klf4	Odontogenic (+) (Sun et al., 2019)
TET1	DNA demethylation		FAM20C	Odontogenic (+) (Li et al., 2018a)
TET3	DNA demethylation			Odontogenic (?) (Argaez-Sosa et al., 2022)
METTL3	RNA methylation		NFIC	Odontogenic (+) (Sheng et al., 2021)

(+): positive effect; (−): negative effect; (?): no clear conclusion yet.

The fate of DPSCs is also influenced by DNA demethylases, which regulate DNA methylation dynamics alongside DNA methyltransferases. The TET family of demethylases, including TET1, TET2, and TET3, plays a key role in this regulation. TET1 promotes odontoblast differentiation in DPSCs while inhibiting adipogenic and osteogenic differentiation. (Wiehle et al., 2016; Cakouros et al., 2019; Qian et al., 2021). As a DNA dioxygenase, TET1 is present in both the nucleus and cytoplasm of DPSCs, and its expression increases during odontogenic induction. However, TET1 expression decreases during early cell passage (less than six generations) (Li et al., 2014). Knockdown of TET1 inhibits both proliferation and odontogenic differentiation (Rao et al., 2016). Additionally, TET1 can promote odontogenic differentiation by regulating FAM20C demethylation, thereby upregulating FAM20C expression (Li et al., 2018a). Interestingly, TET3 is expressed gradually inPLSCs during adipogenic induction, suggesting a tissue-specific role in regulating PLSC cell fate by enhancing adipocyte differentiation. While TET3 may also play a part in the odontogenic differentiation of DPSCs, further research is needed to validate this hypothesis through extensive experiments (Argaez-Sosa et al., 2022).

Reparative dentinogenesis is a delicate balance between odontogenic differentiation and inflammation. DNA methylation plays a role in the inflammatory responses that occur in human dental pulp. During lipopolysaccharide (LPS)-induced inflammation, DNMT1 mRNA and protein levels decrease in DPSCs. DNMT1 influences the promoter methylation of the MyD88 gene and suppresses the expression of miR-146a-5p. Reduction in DNMT1 can activate the NF-κB pathway, thereby intensifying the inflammatory response (Meng et al., 2019; Mo et al., 2019). LPS-treated DPSCs exhibit elevated expression of pro-inflammatory cytokines such as GM-CSF, MCP-2, RANTES, IL-8, and IL-6. Additionally, 5-Aza-CdR activates the NF-κB and MAPK signaling pathways by decreasing the methylation level of the TRAF6 promoter in DPSCs. These findings suggest that 5-Aza-CdR promotes inflammation by activating TRAF6 (Feng et al., 2019). TET2 also modulates inflammation in dental pulp tissue by converting 5-methylcytosine to 5-hydroxymethylcytosine, which promotes DNA demethylation. Evidence suggests that TET2 enhances LPS-induced inflammation by regulating MyD88 hydroxymethylation in an *in vitro* culture model (Wang et al., 2018a). These results imply that DNA methylation plays a crucial role in promoting DPSCs’ odontogenic differentiation and the formation of reparative dentin both *in vitro* and *in vivo*.

In conclusion, further investigation is necessary to uncover the intricate mechanisms governing the methylation-dependent regulation of DPSCs. Such efforts will pave the way for using DPSCs in regenerative tissue bioengineering and vital pulp therapy with optimal safety and efficacy.

### 4.2 Histone modifications associated with odontogenic differentiation in DPSCs

#### 4.2.1 Histone methylation

Histone methylation, a well-researched form of histone modification, involves the methylation of lysine or arginine residues in histone tails and is dynamically regulated by enzymes that control histone methylation and demethylation (Shen et al., 2017) (Table 2). A comparison of the epigenetic status between dental follicle cells (DFCs) and DPSCs showed that H3K27me3-mediated suppression of dental-derived genes DMP1 and DSPP in DFCs was not observed in DPSCs. It has been demonstrated that DPSCs exhibit higher expression of DMP1 and DSPP under osteoinductive conditions (Gopinathan et al., 2013), which is partially attributed to differences in histone methylation profiles. The histone methyltransferase EZH2, which methylates H3K27me3, decreases in activity during odontogenic differentiation, leading to lower levels of H3K27me3. Although a lack of methyltransferase activity in EZH2 was initially not expected to impact DPSCs’ differentiation into odontogenic cells, it has been shown to negatively affect this process. siEZH2 transfection of β-TCP/DPSCs implanted subcutaneously in nude mice has been observed to promote mineralized tissue formation. Additionally, chromatin immunoprecipitation (ChIP) analysis revealed that EZH2 downregulated β-catenin expression by increasing H3K27me3 methylation levels in the β-catenin promoter region, thereby inhibiting the Wnt/β-catenin signaling pathway and suppressing odontogenic differentiation (Li et al., 2018b). Histone methylation of H3K9 plays a role in the osteogenesis of DPSCs. Euchromatin Histone Methyltransferase-1 (EHMT1), an enzyme that represses transcription, demethylates H3K9, thereby promoting cellular differentiation (Kramer, 2016). During osteogenesis stimulated by BMP-2, the co-repressor CBFA22T reduces H3K9me methylation at the Runx promoter, which plays a key role in cellular mineralization and can inhibit H3K9me2 normally mediated by Euchromatin Histone Methyltransferase 1 (EHMT1) at the RUNX2 promoter. Downregulation of CBFA2T2 increases EHMT1 expression, leading to an increase in H3K9me2 methylation and subsequent inhibition of osteogenic differentiation (Huang et al., 2018).

**TABLE 2:** Histone modifications in DPSCs.

Epigenetic modifier	Epigenetic mark	Downstream targets	Epigenetic targets	Result
EZH2	Histone methylation	Wnt/β-Catenin pathway, IL-6,IL-8, CCL2	H3K27me3	Inflammation (+), Odontogenic (+) (Li et al., 2018b)
EHMT1	Histone methylation	Runx2	H3K9me2	Odontogenic (−) (Huang et al., 2018)
MLL	Histone methylation	Wnt5a	H3K4me3	Odontogenic (−) (Shi, 2007)
KDM2A	Histone demethylation	EREG	H3K4/H3K36	Odontogenic (−) (Dong et al., 2013)
KDM6B/JMJD3	Histone demethylation	WNT5A, BMP2 NF-κB	H3K27me3	Odontogeni (+) (Hoang et al., 2016)
KDM5A	Histone demethylation	DMP1, DSPP, OSX,OCN	H3K4me3/me2	Odontogenic (−) (Li et al., 2020)
KDM7A/JHDM1D	Histone demethylation	Wnt/β-catenin	H3K9/27me2	Odontogenic (−) (Huang et al., 2010)
p300	Histone acetylation	OCN, NANOG, SOX2, DSPP, Dmp2, Osx	H3K9ac	Odontogenic (+) (Wang et al., 2014; Liu et al., 2015b)
HDAC6	Histone deacetylation			Odontogenic (+) (Wang et al., 2018b)
HDAC3	Histone deacetylation	Dmp1, Sp7	H3K27ac	Odontogenic (+) (Tao et al., 2020)
TSA	HDACis	DMP1, DSPP, BSP	HDAC 1, 2, 3; HDAC 4,5,6	Odontogenic (+) (Jin et al., 2013)
VPA	HDACis	BSP, OPN	HDAC 1, 2, 3; HDAC 4,5,6	Odontogenic (+) (Paino et al., 2014)
SAHA	HDACis	Nfic, Dspp, Alp, Dmp1, nestin	HDAC1,2,3,8,HDAC4,5,6,7,9,10	Odontogenic (+) (Kwon et al., 2012)
LMK-235	HDACis	ALP, DSPP	HDAC 4, 5	Odontogenic (+) (Liu et al., 2018a)
MS-275	HDACis	RUNX2, DMP1, ALP, DSPP	HDAC 1, 3	Odontogenic (+) (Sultana et al., 2021)

(+): positive effect; (−): negative effect.

Histone methylation is a reversible process regulated by demethylases. KDM2A, a histone demethylase, inhibits the osteogenic and odontogenic differentiation of dental DPSCs by demethylating H3K4 or H3K36, which suppresses the expression of genes such as BSP, OPN, and OCN (Du et al., 2013). Du et al. (2013) found that KDM2A initiates its demethylation activity by binding to the BCL6 co-repressor and targeting the promoter of the epiregulin (EREG) gene, reducing the methylation of H3K4 and H3K36 and thus inhibiting EREG expression, further suppressing osteogenic and odontogenic differentiation in dental-derived mesenchymal stem cells. Dong et al. (2013) observed that using shRNA to interfere with KDM2A expression in dental papilla stem cells resulted in significant increases in stemness-related genes such as SOX2 and H3K4me3 in the promoter region of NANOG. This led to an increase in SOX2 and NANOG gene expression, promoting lipogenic and chondrogenic differentiation. The Jumonji domain-containing protein D3 (JMJD3), also known as lysine-specific demethylase 6B (KDM6B), is a protein that specifically removes H3K27me2/3 methyl marks, thereby regulating gene expression and odontogenic differentiation through multiple mechanisms. Increased expression of JMJD3 enhances odontogenic differentiation, whereas its suppression through the addition of alcohol to the mineralization induction medium inhibits odontogenesis (Hoang et al., 2016). JMJD3 removes the H3K27me3 methylation mark by binding to the BMP2 promoter during odontogenic induction, thus activating the expression of transcriptional proteins associated with BMP2 and odontogenic differentiation (Xu et al., 2013). The promoter region of the Wnt5a gene contains a “bivalent domain” with both repressive H3K27me3 and active H3K4me3 markers. These modifications maintain the Wnt5a gene in a state of balance, allowing its transcription to be either repressed or activated depending on various stimuli (Bernstein et al., 2006). JMJD3 erases H3K27me3 on the Wnt5a promoter and activates Wnt5a in DPSCs under odontogenic stimulation. Conversely, JMJD3 deficiency leads to an increase in H3K27me3, which silences Wnt5a and impedes odontogenesis. Additionally, JMJD3 inhibits odontogenesis through interaction with the mixed-lineage leukemia complex (H3K4me3 methyltransferase) (Zhou et al., 2018).

An additional histone demethylase, known as lysine-specific demethylase 5A (KDM5A), is highly specific for the activity marker H3K4me3 (Shi, 2007). Depletion of KDM5A results in increased levels of H3K4me3 at the promoters of odontogenic differentiation genes, such as DMP1, OSX, OCN, and DSPP, suggesting a link between H3K4me3 and odontogenesis (Li et al., 2020). Another histone demethylase, JHDM1D, also referred to as KDM7A, is used to remove dimethyl tags at lysine nine and lysine 27 on histone H3 of target gene promoters (Huang et al., 2010; Yokoyama et al., 2010). According to a report by Yang et al. (2019), JHDM1D may inhibit osteogenesis in mouse bone marrow cells (ST2) by deactivating the Wnt signaling pathway. JHDM1D knockdown leads to increased mineralized nodule formation in human dental pulp-derived stem cells (hDPSCs) and upregulation of β-catenin expression, which is achieved by downregulating DKK1. Above results suggest that inhibiting JHDM1D expression could activate the Wnt/β-catenin signaling pathway to regulate odontogenesis in hDPSCs.

Furthermore, it has been discovered that modifications to histone methylation are implicated in the pulpal inflammatory response (Hui et al., 2017). Moreover, EZH2 has been shown to serve a key role in the inflammation and regeneration of dental pulp tissue (Hui et al., 2018). Upon TNF-α stimulation, DPSCs differentiate into dentin-producing odontoblasts and migrate to the site of infection to generate reparative dentin. Studies using immunohistochemistry and immunofluorescence have shown that infected cells exhibit significant reductions in EZH2 and H3K27me3 levels. Inhibiting EZH2 downregulates inflammatory factors such as IL-1β, IL-6, and IL-8, which suppresses DPSC proliferation but enhances osteogenesis. This suggests that EZH2 enhances inflammation and cell proliferation while suppressing osteogenic differentiation (Hui et al., 2014). Given that histone methylation is a reversible epigenetic modification, understanding its regulatory mechanism in DPSCs offers the potential to use histone methylation inhibitors in vital pulp therapy to modulate the inflammatory process.

#### 4.2.2 Histone acetylation

The process of histone acetylation is under the control of HAT and HDAC enzymes (Marmorstein and Zhou, 2014), and it has profound consequences on the growth and specialization of various cells (Clayton et al., 2006; Fukuda et al., 2006; Cameron et al., 2016) (Table 2). HAT enzymes can enhance the odontogenesis of DPSCs by increasing histone H3 acetylation on the DSPP gene (Gu et al., 2013). For instance, the HAT enzyme p300, which is a member of the lysine acetyltransferase three family, transfers acetyl groups to lysine residues on histones. p300 predominantly targets the promoter regions of the OCN and DSPP genes, acetylating histone H3K9 and thereby promoting gene transcription and enhancing mineralization and differentiation in dental pulp cells (Wang et al., 2014; Liu et al., 2015b). The overexpression of p300 increases H3K9ac labeling on the OCN and DSPP genes, and it also regulates the promoter regions of NANOG and SOX2, ensuring sufficient expression of stemness-related genes and maintaining stemness (Liu et al., 2015b). Knocking down p300 in DPSCs inhibits proliferation and odontogenic differentiation, consistent with a previous report (Haigis and Guarente, 2006). HDAC enzymes also play a role in histone acetylation by removing acetyl groups from histones and facilitating the densification of chromatin structure (Fukuda et al., 2006). There are 18 known human HDACs, categorized into four classes based on homology to yeast HDACs. Classes I (HDAC1, HDAC2, HDAC3, HDAC8), II (HDAC4, HDAC5, HDAC6, HDAC7, HDAC9, HDAC10), and IV (HDAC11) are zinc-dependent enzymes with high sequence conservation (Wang et al., 2018b). Also, Class III HDAC (usually known as sirtuins) is a nicotinamide adenine dinucleotide-reliant enzyme (Haigis and Guarente, 2006). HDAC6 was found to promote the DPSCs odontogenic differentiation, and the mineralization and ALP activity capability in pDPSCs were increased when HDAC6 was knocked down (Wang et al., 2018b). In mouse tooth papillary mesenchymal cells subjected to odontoblast induction, elevated p300 expression and decreased HDAC3 expression were observed, leading to an increase in H3K9ac and H3K27ac levels. This suggests that HATs and HDACs regulate odontogenic specialization in a coordinated manner (Tao et al., 2020). For further exploration, the transcription factor KLF4 is found to have a trans-activation domain that directly attaches to target gene promoters, recruiting either co-repressors like HDAC3 or coactivators like p300 (Tao et al., 2019). Chromatin immunoprecipitation (ChIP) assays have shown that HDAC3 and p300 regulate odontogenic differentiation in a time-sensitive manner through interactions with KLF4. When dental pulp cells are induced into odontoblasts, HDAC3 primarily interacts with KLF4 at the Sp7 and Dmp1 promoters on the first day of induction, keeping DMP1 and OSX expression at moderate levels. However, on day 7, HDAC3 relocates to the cytoplasm, allowing KLF4 to link with p300 and transactivate Dmp1 and Osx, thereby enhancing odontoblast differentiation. These findings reveal that KLF4 can influence histone acetylation in the promoter regions of both Sp7 and Dmp1, regulating odontoblast differentiation through interactions with HDAC3 and p300 (Tao et al., 2019).

It has been documented that HDAC inhibitors (HDACi) have the potential to modulate gene expression through the regulation of histone acetylation levels. These inhibitors have been studied for their therapeutic potential in cancer treatment (Lakshmaiah et al., 2014), inflammatory diseases (Hull et al., 2016), and neurodegenerative disorders (Didonna and Opal, 2015). These inhibitors also influence the differentiation and proliferation of DPSCs, showing promising prospects for pulp regeneration. Pan-HDAC inhibitors such as Trichostatin A (TSA), suberoylanilide hydroxamic acid (SAHA), and valproic acid (VPA) hold potential for use in dental restorations (Duncan et al., 2011). Recent research demonstrates that TSA, an isohydroxamic acid, can inhibit the activity of all HDACs except class IIA, enhancing the expression of proliferating cell nuclear antigen (PCNA), cell cycle-related protein CCDN1, DSPP, DMP1, and BSP, thus promoting mineralization and differentiation of DPSCs (Jin et al., 2013). The Smad2 and Smad3 signaling pathways play a key role in mediating mineralization and differentiation of DPSCs stimulated by TSA. Moreover, recent *in vivo* studies have explored the developmental effects of HDAC inhibitors on dental pulp by injecting pregnant mice with TSA. Compared to control groups, an increase of 1.64-fold in the dentin matrix and 1.74-fold in the number of odontoblasts was observed, suggesting that histone acetylation plays a critical role in mouse tooth development (Jin et al., 2013). VPA, a short-chain fatty acid, can inhibit class I HDACs. Research suggests that low concentrations of VPA promote DPSC mineralization and correlate with increased expression of osteopontin (OSP) and bone sialoprotein (BSP) (Paino et al., 2014). VPA heightens the expression of OPN and BMP through HDAC2 while simultaneously reducing OCN, a belated marker of osteogenic differentiation. This suggests that VPA enhances early osteogenic differentiation, but not the terminal differentiation phase. Gene expression levels were accompanied by the suppression of another class I HDAC, HDAC-2 (Paino et al., 2014). SAHA, a type of pan-inhibitor of HDACs, activates Nfic, increasing DSPP expression and thus enhancing the DPSCs odontogenesis (Kwon et al., 2012). From a mechanistic perspective, SAHA was recently discovered to encourage matrix metalloproteinase 13 (MMP-13) expression in rat DPSCs to promote mineralization and migration. Despite this, low concentrations of SAHA did not affect cell proliferation (Duncan et al., 2016). As a means of reducing off-target effects and improving specificity over conventional pan-HDAC inhibitors, the development of subtype-specific HDACs has been underway for about a decade (Balasubramanian et al., 2009). A recent study demonstrated that LMK-235, a selective inhibitor of HDAC-4 and HDAC-5, enhances the differentiation of human DPSCs into odontoblast-like cells while having no effect on cell proliferation (Liu et al., 2018a). Similarly, MS-275 is a specific HDACi that targets both HDAC1 and HDAC3. Administration of MS-275 in normal cultures of pDPSCs can upregulate the expression of ALP, RUNX2, DMP1, and DSPP without being cytotoxic at a concentration of 20 nmol/L. Remarkably, the MAPK signaling system remains largely inactive upon MS-275 stimulation, indicating that it is unlikely that MAPK signaling pathway transduction is involved in MS-275-induced odontogenesis (Lee et al., 2020). The odontogenic potential of MS-275 has also been observed in MDPC-23 cells, a murine odontoblast-like cell line, where MS-275 alone can enhance the expression of Runx2, Bmp2, Bmp4, Ocn, Dspp, Dmp1, Klf5, Col1α1, and Msx1, and increase calcified nodule formation and ALP activity without mineralized medium induction (Sultana et al., 2021). These findings support the use of target-specific HDACis to promote odontoblast differentiation, thereby creating a promising avenue for potential therapeutic applications (Manaspon et al., 2021). Histone acetylation regulates a variety of physiological processes in DPSCs, which can impact their fate. While there is still much to be explored, As a potential mineralized regeneration tool, HDACis may hold promise.

### 4.3 ncRNAs associated with odontogenic differentiation in DPSCs

#### 4.3.1 MiRNAs

The field of epigenetics has seen a surge of interest in miRNAs, with numerous studies on their role in the odontogenic differentiation of DPSCs(Table 3). Using PCR analysis of 104 known miRNAs, 48 were differentially expressed between DPSCs and BM-MSCs, with 17 upregulated and 29 downregulated (Vasanthan et al., 2015). High-throughput microarray analysis revealed that 22 miRNAs were differentially expressed in odontogenic differentiated DPSCs, with 12 upregulated and 10 downregulated. It is of particular interest to note that miR-135b was significantly downregulated in the course of pulp cell mineralization. In light of the similarity between odontogenesis and osteogenesis and miR-135b’s significance in osteogenesis, it is postulated that miR-135b may serve a key part in regulating differentiated DPSCs mineralization (Schaap-Oziemlak et al., 2010). A recent study targeting miR-135b revealed its ability to inhibit odontogenic differentiation of DPSCs by modulating both SMAD5 and SMAD4 (Song et al., 2017). The miR-143 family exerts a negative regulatory effect on the odontogenic and osteogenic differentiation of DPSCs. Specifically, miR-143-5p is known to target RUNX2 to impair odontogenic differentiation through the OPG/RANKL pathway (Zhan et al., 2018). A number of studies have shown that miR-143-5p interacts with MAPK14, thereby reducing its expression. This leads to an induction of MAPK14 expression upon miR-143-5p knockdown, activating the p38 MAPK signaling pathway and enhancing odontogenesis (Wang et al., 2019a). Additionally, microarray analyses have revealed that miR-143 and miR-145 are significantly downregulated in mouse DPSCs and can form a negative regulatory feedback loop with KLF4 to inhibit the expression of Dspp and Dmp1 mRNA and protein, consequently having a negative impact on odontogenic differentiation (Liu et al., 2013). Importantly, this represents the first report of a feedback loop regulation involving miRNA during odontogenic differentiation.

**TABLE 3:** miRNAs-mediated regulation of odontogenic differentiation in DPSCs.

ncRNAs	Targets	Differentiation
miR-135b	SMAD5/SMAD4	Odontogenic (−) (Song et al., 2017)
miR-143-5p	Runx2, MAPK14, TNF-α	Odontogenic (−) (Zhan et al., 2018; Wang et al., 2019a)
miR-145	KLF4	Odontogenic (−) (Liu et al., 2013)
miR-140-5p	Wnt1/β-catenin	Odontogenic (−) (Lu et al., 2019)
let-7c, let-7c-5p	IGF-1R, DMP1	inflammation Odontogenic (−) (Liu et al., 2018b)
miR-218	RUNX2	Odontogenic (−) (Chang et al., 2019)
miR-431		Odontogenic (−)
miR-508-5p	GPNMB	Odontogenic (−) (Liu et al., 2019)
miR-488	p38 MAPK	Odontogenic (−) (Yu et al., 2019)
miR-720	NANOG	Odontogenic (+) (Hara et al., 2013)
miR-146a-5p		Odontogenic (+) (Qiu et al., 2019)
miR-675	DLX3	Odontogenic (+) (Zeng et al., 2018a)
miR-27a-5p	TGF-β1/smads	Odontogenic (+) (Hu et al., 2019)
miR-223-3p	Smad3	Odontogenic (+) (Huang et al., 2019)
miR-506		inflammation (Wang et al., 2019b)
miR-125a-3p	Fyn	Odontogenic (+) inflammation (Wang et al., 2020)
miR-21	STAT3	Odontogenic (+) (Xu et al., 2018a)

(+): positive effect; (−): negative effect.

Various other miRNAs have been affiliated with the DPSCs osteogenic and odontogenic differentiation. Upon induction of DPSCs into odontoblasts, miR-140-5p is decreased, and miR-140-5p mimics can impede odontogenic differentiation by targeting Wnt1 to repress the Wnt1/β-catenin signaling pathway (Lu et al., 2019). During odontogenesis induction, a gradual decline in miR-508-5p and a concomitant increase in glycoprotein non-metastatic melanoma protein B (GPNMB) can also be observed. Subsequent investigations have demonstrated that the knockdown of miR508-5p, which releases GPNMB expression, bolsters odontogenesis in DPSCs (Liu et al., 2019). In DPSCs, according to Chang et al. (2019), miR-218 expression is differentially downregulated, and miR-218 mimics considerably decrease DPSC proliferation and differentiation. It has also been established that insulin-like growth factor 1 induces DPSCs proliferation and osteogenic/odontogenic differentiation via activating the JNK and P38MAPK pathways. Increasing let-7c inhibits the insulin-like factor-1 receptor (IGF-1R) by reversing the process as well as inhibiting activation of the JNK/P38 MAPK pathway (Liu et al., 2018b). This process, including miR-488, may also be adversely involved (Yu et al., 2019).

In addition to their inhibitory effects, miRNAs can promote odontogenic differentiation in DPSCs (Sun et al., 2015). Studies have shown that miR-720 can promote odontogenic differentiation while concurrently reducing the proliferative capacity of DPSCs. As such, miR-720 represents a novel miRNA involved in the process of DPSCs differentiation (Hara et al., 2013). Kato (2018) demonstrated that lentiviral transfection of hDPSCs with miR-146a-5p resulted in its overexpression, which led to a significant upregulation in the expression of odontogenic differentiation markers (Dspp and Dmp1), suggesting a positive regulatory role of miR-146a-5p in odontogenic differentiation of hDPSCs. Through the inhibition of DNMT3B, miR-675 promotes the odontogenic differentiation of DPSCs by mediating DLX3 methylation (Qiu et al., 2019). Another miRNA, miR-27a-5p, positively regulates odontogenic differentiation by suppressing the LTBP1 molecule and activating the TGF-β1/Smad pathway (Hu et al., 2019).

Interestingly, this miRNA is found in the exosomes secreted by DPSCs during odontogenic induction. There is a conserved motif in miRNA, which can be recognized by RNA-binding proteins and transported in a cell-specific manner. When miRNA is present in the EXOmotif, it is recognized by specific RNA-binding proteins, loaded into vesicles, transported to other cells, and recognized by other cells for cell interaction; miRNA in vesicles can also inhibit gene expression in the receiving cells (Garcia-Martin et al., 2022). In the future, RNA editing technology can be used to specifically change the motif of specific miRNA molecules and solve the problem caused by disordered cell interaction, opening up new ideas for future treatment in the oral field.

The modulation of miRNA is an essential aspect during the development of dental pulp inflammation, and studies have identified its distinct expression profiles in healthy and inflamed dental pulp (Zhong et al., 2012). The odontoblast differentiation of pDPSCs can be stimulated by LPS or TNF-α (He et al., 2015). In most cases, the protective effect of miRNA is achieved by mitigating the inflammatory response or promoting odontogenic differentiation. A significantly increased miRNA in inflamed pulp tissue is miR-223-3p, which can be detected in pulp tissue. Overexpression of miR-223-3p has been found to promote DPSCs odontoblast differentiation *in vitro* (Huang et al., 2019). Both let-7c-5p and miR-506 can safeguard against LPS-induced pDPSC inflammation by reducing the pro-inflammatory cytokines performance (Wang et al., 2019b; Huang et al., 2019). *In vivo* experiments have confirmed that let-7c-5p reduces the ability of LPS to induce pulpitis in rats (Yuan et al., 2018). Furthermore, let-7c-5p has additional potential for promoting bone growth in inflamed pDPSCs(Yuan et al., 2019). Knocking down miR-140-5p has been found to enhance odontoblast differentiation, and Toll-like receptor-4 is involved in the miR-140-5p-mediated effects on pDPSCs, inhibiting pDPSC proliferation upon LPS stimulation (Sun et al., 2017). Fyn is a member of the Src kinase family, which has been reported to be upregulated in the microenvironment of deep caries. The miR-125a-3p has been identified as an upstream factor of Fyn and a positive factor in regulating pDPSC odontoblast differentiation upon TNF-αstimulation (Wang et al., 2020). At low concentrations (1–10 ng/mL), TNF-αenhances odontogenic differentiation, while at high concentrations, it suppresses it. As shown above, miR-21 is detected even at low levels of TNF-α, along with an increase in the expression of signal transduction and activator of transcription3 (STAT3). When high concentrations are present, the opposite results are observed. STAT and miR-21 are both involved in positive feedback loops regulating odontogenic differentiation (Xu et al., 2018a).

**FIGURE 3:**
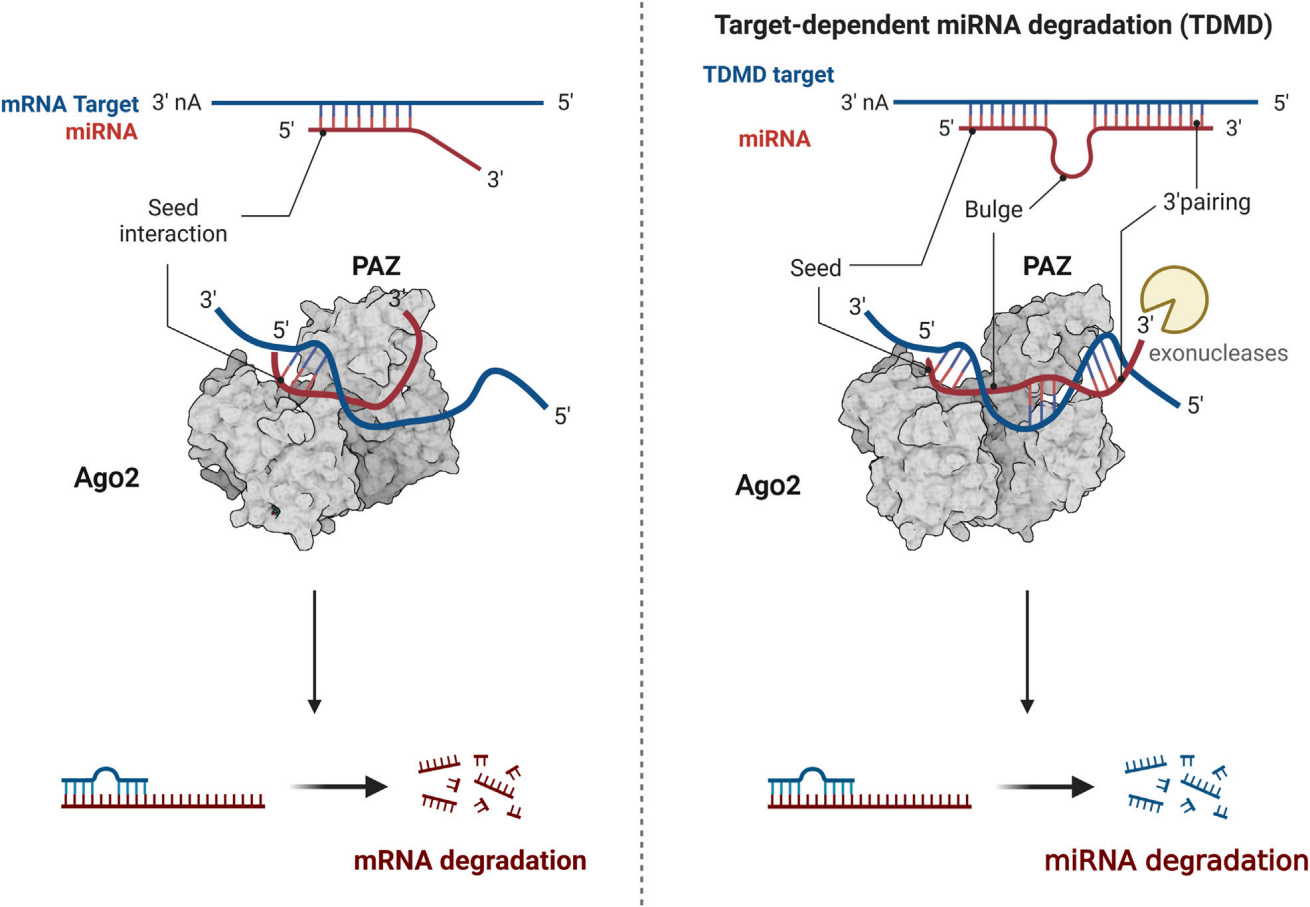
The modulation of TDMD mechanism. The 3′end of miRNA does not fully bind to target genes, miRNA remains relatively stable. The 3′end of miRNA can fully bind to target genes, miRNA is easily degraded. Created with BioRender.com.

MiRNA, as a subject of epigenetic research, has attracted much attention, with many theories constantly being updated and improved. One such theory is target-directed miRNA degradation (TDMD), where target genes can degrade miRNA by binding to it (Figure 3). The form of miRNA and target gene binding influences the stability of miRNA. When the 3′end of miRNA does not fully bind to target genes, miRNA remains relatively stable. However, miRNA is easily degraded when the 3′end of miRNA can fully bind to target genes (Kato, 2018). For instance, research has shown that the 3′end of miR-382-3P can be completely complementary to the upstream gene GAS5, leading to the autodegradation of miR-382-3p. This process relieves the inhibitory effect of the downstream target gene TAF1, promoting the osteogenic differentiation of hBMSCs (Song et al., 2022). Another emerging concept is the nuclear activating miRNA (NamiRNA), which investigates the epigenetic regulatory mechanisms of miRNAs themselves. A systematic analysis of 1,594 miRNA precursors across seven different tissue types revealed more than 300 miRNA precursors with genomic positions overlapping enhancer histone modification markers H3K4me1 or H3K27ac. This association between tissue-specific miRNA and enhancers led to the discovery that the activation of these nuclear miRNAs relies on the integrity of the enhancer region. Knocking down specific sequences of the enhancer can prevent miRNA upregulation, affecting tumor cells’ proliferation and migration capabilities. Some scholars suggest that miRNAs function as bifunctional molecules. In the cytoplasm, miRNAs act as negative regulators, blocking mRNA translation and suppressing gene expression. Conversely, in the nucleus, miRNAs serve as activators, modulating the chromatin state of enhancers to stimulate gene transcription (Xiao et al., 2017).

While these theories have yet to be applied to the odontogenic differentiation of DPSCs, they present innovative concepts and perspectives that could stimulate scientific inquiry and inspire further research into restorative dentin and pulp regeneration. These ideas offer potential pathways for more in-depth exploration of these fields, potentially leading to breakthroughs in regenerative dental therapies.

#### 4.3.2 LncRNAs

A mounting body of research underscores the significant regulatory role of lncRNA in the differentiation process of hDPSCs(Table 4). One such lncRNA, H19, is an imprinted paternal gene that encodes a 2.3-kb noncoding RNA and exhibits multiple biological effects, including promoting the ability of hDPSCs proliferation, migration, and differentiation (Huang et al., 2016). Research indicates that H19 inhibits the expression of LATS1 by increasing zeste homolog two-induced trimethylation levels of histone three at lysine 27. Conversely, LATS1 suppresses the differentiation, proliferation, and migration of hDPSCs by decreasing YAP and TAZ levels, thereby activating the Hippo-Yes-related protein (YAP) signaling pathway. Transplanting hDPSCs overexpressing lncRNA H19 into nude mice showed that lncRNA H19 inhibits LATS1 and promotes the production of odontoblast cells *in vivo* (Du et al., 2021). H19 also upregulates S-adenosyl homocysteine (SAH), an inhibitor of S-adenosylmethionine-dependent methyltransferase (Zhou et al., 2015). It enhances the odontogenic differentiation of DPSCs by downregulating the methylation level of the distal-free homology cassette 3 (DLX3) gene to upregulate the expression of DLX3 (Zeng et al., 2018b). Furthermore, H19 can bind to miR-140-5P via the sponge mechanism, which derepresses miR-140-5P on the downstream edentulous target genes (BMP-2, FGF9) to promote odontogenic differentiation (Zhong et al., 2020). Nonetheless, research has shown that lncRNA H19 expression was not significantly detected during odontogenesis. This discrepancy may arise from the fact that differentiated and undifferentiated cells used in the studies were derived from different individuals, potentially leading to varying results. Additionally, phenotypic differences and variations in sequencing methods may introduce errors. Therefore, large-scale studies are required to confirm these findings and provide more definitive conclusions (Zhong et al., 2020). In addition to chromosomal modifications that participate in epigenetic regulation, long non-coding RNAs (LncRNAs) may also engage with the key molecules of related signaling pathways to regulate the odontogenic differentiation of DPSCs. The LncRNA anti-differentiation non-coding RNA (ANCR), also known as differentiation antagonistic non-protein coding RNA (DANCR), was initially discovered in 2012 to inhibit differentiation and promote undifferentiation of somatic progenitor cells (Kretz et al., 2012). Furthermore, studies have revealed that inhibition of ANCR leads to neurogenic, osteogenic, and lipogenic differentiation in DPSCs without impeding their proliferation (Jia et al., 2016). ANCR impedes the odontogenic differentiation of DPSCs by targeting the Wnt/β-catenin signaling pathway (Chen et al., 2016).

**TABLE 4:** LncRNAs-mediated regulation of odontogenic differentiation in DPSCs.

ncRNAs	Targets	Differentiation
H19	SAHH/miR-140-5P	Odontogenic (+) (Zhou et al., 2015; Zeng et al., 2018b; Zhong et al., 2020)
DANCR	p-GSK-3β/β-catenin	Odontogenic (−) (Chen et al., 2016)
G043225	miR-588	Odontogenic (+) (Chen et al., 2020)
MALAT1	miR-140-5p/GIT2	Odontogenic (+) (Bao et al., 2020)
SNHG1	miR-328-3p/TGF-β/Wnt	Odontogenic (−) (Fu et al., 2022)
SNHG7		Odontogenic (+) (Liu et al., 2020)

(+): positive effect; (−): negative effect.

The ability of dental pulp stem cells (DPSCs) to undergo odontogenic differentiation has been associated with the activity of lncRNAs. RNA-sequencing analysis has revealed a correlation between the loss of odontogenic differentiation potential and the downregulation of 108 lncRNAs, as well as the upregulation of 36 lncRNAs (Zheng and Jia, 2016). Moreover, inducing odontoblastic differentiation in DPSCs differentially alters the expression of 132 lncRNAs and 114 miRNAs. Bioinformatic analysis has identified lncRNAs linked to odontogenic proteins such as retinoid and fibronectin 1. Their associated competing endogenous RNA (ceRNA) networks play a role in the complex differentiation of DPSCs. Further studies have shown that lncRNA G043225 inhibits the expression of FBN1 by acting as an endogenous miRNA sponge, competitively inhibiting miR-588, thereby promoting odontogenic differentiation (Chen et al., 2020).

Prominent researchers have meticulously investigated the novel regulatory circuit governing the odontogenic differentiation of hDPSCs. This circuit involves an interplay between the long non-coding RNA metastasis-related lung adenocarcinoma transcript 1 (MALAT1), microRNA-140-5p (miR-140-5p), and the G protein-coupled receptor kinase two interaction protein 2 (GIT2). During the odontogenic differentiation of hDPSCs, miR-140-5p is significantly downregulated. Dual-luciferase reporter gene assays have demonstrated that miR-140-5p directly targets GIT2. RNA pull-down assays have further confirmed that MALAT1 can bind to miR-140-5p, thereby positively regulating GIT2 expression. Notably, increased miR-140-5p levels or GIT2 knockdown in hDPSCs (via exogenous transfection or lentiviral infection) inhibits the expression of key markers of odontogenic differentiation, such as ALP activity, DSPP, DMP-1, and DLX3. Collectively, these findings suggest that MALAT1 may play a crucial role in regulating odontogenic differentiation in hDPSCs (Bao et al., 2020). Analogously, the lncRNA colon cancer-associated transcript 1 (CCAT1) was initially found to participate in various aspects of metabolism, migration, and proliferative processes in select cancers (Li et al., 2018c). Subsequently, researchers have established that CCAT1 can also promote the DPSCs proliferation of odontogenic differentiation and proliferation. Remarkably, luciferase assays have evinced that CCAT1 is bound to miR-218, leading to negative modulation of miR-218 expression (Zhong et al., 2019). The most recent investigation has unveiled that the impact of small nucleolar RNA host gene 1 (SNHG1) can influence the progression of osteosarcoma by controlling miR-101-3p, miR-326, and miR-577 (Wang et al., 2018c; Jiang et al., 2018; Deng et al., 2019). Furthermore, SNHG1 can also impede the proliferation of colorectal cancer cells through miR-154-5p (Xu et al., 2018b). The overexpression of SNHG1 is observed during the differentiation of hDPSCs into odontoblast-like cells. SNHG1 expression increases progressively on days 0, 3, and seven of hDPSCs’ odontogenic differentiation. The overexpression of SNHG1 boosts the mRNA and protein expression of DSPP, DMP-1, and ALP. Mechanistically, SNHG1 can bind to miR-328-3p, lifting its inhibitory effect on target genes to activate the Wnt/β-catenin pathway, thereby promoting odontogenic differentiation (Fu et al., 2022). Recent research has highlighted substantial alterations in SNHG7 expression during the odontogenic differentiation of DPSCs. By culturing human DPSCs in an osteogenic/odontogenic differentiation medium for 14 days and analyzing the cells’ RNA sequences, scientists identified 89 differentially expressed lncRNAs, 1,636 mRNAs, and 113 miRNAs. Silencing SNHG7 was found to inhibit the odontogenic/osteogenic differentiation of DPSCs, suggesting it as a promising target for dentin-pulp complex regeneration and tissue engineering (Liu et al., 2020).

Despite progress, significant knowledge gaps remain in understanding the role of lncRNAs in odontogenic differentiation. Further research is needed to confirm the functions of various molecules involved. The current understanding centers around competing endogenous RNA (ceRNA) competition, transcription factor regulation, and DNA methylation interactions. However, more intricate and nuanced epigenetic mechanisms should be explored. It is clear that lncRNAs play a critical role in the differentiation potential of hDPSCs and represent a promising target for new treatments in VPT.

#### 4.3.3 CircRNAs

CircRNA, with its closed-loop system, is highly conserved and extremely resistant to RNA enzymes, making it a noteworthy molecular entity. Moreover, it is recognized as a potent miRNA sponge due to its ability to competitively bind miRNAs. First discovered in viruses in 1976, circRNA has since been identified as a pivotal post-transcriptional regulator of cell proliferation, differentiation, and apoptosis (Wang et al., 2019c).

Numerous studies have verified the significant role of circRNAs in regulating the osteogenic differentiation of dental stem cells. However, there have been few studies on the effects of circRNAs on the odontogenic differentiation of DPSCs. Among them, Chen et al. (2020) have utilized microarray analysis to determine that 187 circRNAs have been differentially expressed more than 1.5-fold during odontogenic differentiation in the induced group compared to the non-mineralized induced group, of which 44 circRNAs have been upregulated, and 143 have been downregulated. In the induced group, hsa_circRNA_005044, hsa_circRNA_005044, hsa_circRNA_406763, and hsa_circRNA_104101 have been upregulated 3.78, 8.29, 15.95, 19.03-fold, respectively, compared to the uninduced group. Furthermore, hsa_circRNA_079813 has undergone a 3.85-fold downregulation and hsa_circRNA_008336 has undergone a 3.74-fold downregulation. Among the 187 circRNAs that have significantly different expressions prior to mineralization induction, these six circRNAs exhibit the most pronounced changes. Knockdown of circRNA_104101 has resulted in varying degrees of reduction in odontogenic markers, such as ALP, DSPP, DMP1, and OCN, after the 7th and 14th days of induction. Furthermore, based on bioinformatics analysis, it is indicated that the Wnt/TGF-β signaling pathway may be involved; however, the precise mechanism still requires further exploration. Li and Jiang (2019) have identified 1314 upregulated and 1780 downregulated circular RNAs, respectively, during odontogenic induction using high-throughput sequencing. The expression trends obtained by high-throughput sequencing have been consistent with the results of RT-qPCR screening and validation of two circular RNAs with large fold changes (hsa_circ_0015260 and hsa_circ_0006984). Additionally, the MAPK signaling pathway has been predicted to be part of odontogenic differentiation, but further investigation is necessary to determine specific results and mechanisms.

The bulk of research on the odontogenic differentiation of DPSCs has primarily centered on miRNAs, with circRNAs receiving relatively little attention. Given their inherent stability and significant abundance in physiological systems, circRNAs represent a promising avenue for biomarker research related to disease diagnosis (Han et al., 2018). However, numerous molecules and mechanisms remain to be explored in greater depth, with circRNAs presenting as potentially powerful targets for VPT. This paper serves as a concise summary intended to provide a reference point for the continued investigation into the regulatory mechanisms of circRNAs in the odontogenic differentiation of DPSCs.

## 5 Conclusion

This comprehensive review examines the odontogenic differentiation of DPSCs with a focus on the critical role of epigenetic modifications, including DNA methylation, histone modifications, and non-coding RNA regulation. Although most current research emphasizes *in vitro* studies, further *in vivo* research and animal disease models are essential for advancing our understanding. Additionally, the field shows great potential for progress in circRNA research. It is important to highlight two complementary concepts, TDMD and NamiRNA, which enhance our understanding of the unique impacts of miRNA molecules across different cellular locations. Given the wide range of epigenetic modifications and underlying mechanisms, our comprehension of epigenetic regulatory influences remains in its early stages, with a focus on classical epigenetic modifications and their respective locations.

Currently, clinical practice mainly employs biomaterials embedded with HDACi and integrates inhibitors into biologically derived dental restorative biomaterials for pulp restoration in exposed areas. However, advances in biotechnology are leading to the discovery of new epigenetic modifications, such as DNA 6 mA modifications, tRNA-enriched epigenetic modifications, and molecular variant shearing, all of which play roles in regulating odontogenic differentiation in DPSCs.The complex interplay of these various modifications presents a challenge in unraveling the epigenetic regulatory mechanisms that govern the odontogenic differentiation of dental pulp stem cells.

## 6 Future direction

Current trends in dentistry emphasize minimally invasive and biologically oriented restorative strategies, focusing on preserving the vital pulp and harnessing its natural regenerative abilities to facilitate self-renewal. The use of calcium silicate-based materials has increased the success of pulp regeneration therapies. However, limitations persist with existing dental materials, such as significant cytotoxicity, reduced tissue repair capacity, and a lack of target specificity. A comprehensive understanding of the biological mechanisms of pulp repair and regeneration is crucial for advancing treatment options. While DPSCs have been employed *in vitro* and in animal models for tissue engineering, their clinical applications remain limited. The expansion and cultivation of DPSCs involve complex procedures that require specialized culture media and conditions to preserve their pluripotency and multipotency. These challenges demand urgent solutions, particularly for large-scale clinical use. Given the variation in the differentiation potential of DPSCs across individuals and sources, patient outcomes may differ.

Looking forward, there are several key areas of focus: 1. Enhancing research on the growth and culture conditions of DPSCs is vital to optimize their expansion and stability while retaining their totipotency and pluripotency; 2. Developing a deeper understanding of the mechanisms and regulatory networks that govern DPSC differentiation is essential. Exploring additional epigenetic processes, such as lactation and succination modifications, can promote targeted differentiation of DPSCs into specific cell lines and improve the efficiency of regenerative therapies; 3. Improving regenerative therapy efficiency through the integration of biomaterials and bioengineering technologies is crucial. By combining these approaches to design suitable scaffolds and carriers, optimal growth environments can be created for DPSCs, promoting their directed differentiation and facilitating dental pulp tissue regeneration and repair; 4. Advances in pharmacology offer opportunities to target essential pulp-preserving therapies against epigenetic or other cellular markers, opening the door to clinical applications of novel endodontic restorative materials. However, the use of epigenetic agents presents several unresolved challenges that warrant attention, including: i. The absence of robust clinical trial data regarding the induction of dentition, raising significant ethical and regulatory concerns; ii. Limited specificity, as inhibitors targeting particular HDAC classes have been developed but lack total specificity, potentially leading to associated side effects; iii. Heavy reliance on the Zn2+ binding motif, which may strongly bind to other vital metalloenzymes, inducing cytotoxicity and restricting the clinical applicability of the inhibitors. Consequently, the future may call for the creation of highly precise inhibitors or the examination of combination therapies to mitigate dose dependency and enhance drug efficacy.
